# Epigallocatechin 3-gallate-induced neuroprotection in neurodegenerative diseases: molecular mechanisms and clinical insights

**DOI:** 10.1007/s11010-025-05211-4

**Published:** 2025-01-20

**Authors:** Md. Rezaul Islam, Abdur Rauf, Sumiya Akter, Happy Akter, Md. Ibrahim Khalil Al-Imran, Samiul Islam, Meherun Nessa, Chaity Jahan Shompa, Md. Nabil Rihan Shuvo, Imtiaz Khan, Waleed Al Abdulmonem, Abdullah S. M. Aljohani, Muhammad Imran, Marcello Iriti

**Affiliations:** 1 Department of Pharmacy, Faculty of Health and Life Sciences, Daffodil International University, Daffodil Smart City, Birulia, Savar, Dhaka, 1216 Bangladesh; 2 Department of Chemistry, University of Swabi, Anbar, 23561 Khyber Pakhtunkhwa Pakistan; 3 Padma View College of Nursing, Dhaka, Bangladesh; 4 Department of Entomology, The University of Agriculture, University of Peshawar, Peshawar, KP Pakistan; 5 Department of Pathology, College of Medicine, Qassim University, Buraydah, Saudi Arabia; 6 Department of Medical Biosciences, College of Veterinary Medicine, Qassim University, Buraydah, Saudi Arabia; 7 Chemistry Department, Faculty of Science, King Khalid University, P.O. Box 9004, 61413 Abha, Saudi Arabia; 8 Department of Biomedical, Surgical and Dental Sciences, University of Milan, Via Luigi Vanvitelli 32, 20133 Milan, Italy; 9 National Interuniversity Consortium of Materials Science and Technology (INSTM), 50121 Florence, Italy

**Keywords:** Epigallocatechin 3-gallate, Neurodegeneration, Molecular mechanisms, Clinical insights, Neuroprotection

## Abstract

Neurodegenerative diseases (NDs) are caused by progressive neuronal death and cognitive decline. Epigallocatechin 3-gallate (EGCG) is a polyphenolic molecule in green tea as a neuroprotective agent. This review evaluates the therapeutic effects of EGCG and explores the molecular mechanisms that show its neuroprotective properties. EGCG protects neurons in several ways, such as by lowering oxidative stress, stopping Aβ from aggregation together, changing cell signaling pathways, and decreasing inflammation. Furthermore, it promotes autophagy and improves mitochondrial activity, supporting neuronal survival. Clinical studies have demonstrated that EGCG supplementation can reduce neurodegenerative biomarkers and enhance cognitive function. This review provides insights into the molecular mechanisms and therapeutic potential of EGCG in treating various NDs. EGCG reduces oxidative stress by scavenging free radicals and enhancing antioxidant enzyme activity, aiding neuronal defense. It also protects neurons and improves cognitive abilities by inhibiting the toxicity and aggregation of Aβ peptides. It changes important cell signaling pathways like Nrf2, PI3K/Akt, and MAPK, which are necessary for cell survival, cell death, and inflammation. Additionally, it has strong anti-inflammatory properties because it inhibits microglial activation and downregulates pro-inflammatory cytokines. It improves mitochondrial function by reducing oxidative stress, increasing ATP synthesis, and promoting mitochondrial biogenesis, which promotes neurons’ survival and energy metabolism. In addition, it also triggers autophagy, a cellular process that breaks down and recycles damaged proteins and organelles, eliminating neurotoxic aggregates and maintaining cellular homeostasis. Moreover, it holds significant promise as an ND treatment, but future research should focus on increasing bioavailability and understanding its long-term clinical effects. Future studies should focus on improving EGCG delivery and understanding its long-term effects in therapeutic settings. It can potentially be a therapeutic agent for managing NDs, indicating a need for further research.

## Introduction

Neurodegenerative diseases (NDs) are characterized by the progressive loss of neurons in specific brain regions [1]. NDs, which significantly contribute to the global disease burden, are a growing global public health concern due to their significant impact [2]. Alzheimer’s disease (AD) is the leading cause of dementia in the senior population and ranks fifth in causes of death. Approximately 44 million people worldwide suffer from it. France currently has 1.1 million AD patients, with 225,000 new cases reported annually. By 2050, the number of patients should nearly double due to population aging [3]. The annual incidence of traumatic spinal cord injury (TSCI) in Finland is 36.6 per million, resulting in 200 new cases annually [4]. With an estimated 64–74 million people suffering a traumatic brain injury (TBI) annually, TBI is a major cause of death and disability and a global public health concern [5]. Huntington’s disease (HD) prevalence in Canada ranges from 4.0 to 13.9 individuals per 100,000 in the general population and 17.2 in the Caucasian population, primarily in British Columbia [6–8]. Based on estimates derived from Fisher and Hayden, there may be as many as 4700 HD cases and 14,000 HD cases at 50% risk in Canada [6]. Multiple sclerosis (MS) is a prevalent chronic ND affecting 2.2 million people globally, primarily affecting young adults [9]. A clinical investigation was conducted to investigate the impact of green tea consumption on cognitive impairment. Twelve elderly nursing home patients with cognitive dysfunction (2 males, 10 women; mean age, 88 years) with a score of less than 28 on the Japanese version of the Mini-Mental State Examination (MMSE-J) took part in the study. The study found that subjects who consumed two grams of green tea powder daily for three months significantly improved their MMSE-J scores [10]. A study in Japan found that daily consumption of 336.4 mg of decaffeinated green tea catechins (GTC) may improve cognitive function. The study involved Japanese adults aged 50–69 with cognitive decline and a MMSE score of > 24. Daily GTC consumption significantly improved cognitive function, suggesting the potential for improved working memory [11]. A meta-analysis of eight studies with 344,895 individuals and seven studies with 492,724 people found a linear link between tea and caffeine use and the risk of PD. Consuming two cups of coffee daily or 200 mg daily can reduce the smoking-adjusted risk of PD by 26 and 17%, respectively [12, 13]. Another study in Japan found that green tea or EGCG did not significantly impact cognitive functioning. Participants were given either 2 g/day of green tea powder or a placebo. Drinking green tea for a year had no discernible impact, but the green tea group had lower levels of malondialdehyde-modified low-density lipoprotein and oxidative stress (OS) markers [14]. A double-blind, placebo-controlled crossover study involved administering 27 healthy individuals with two dosages of EGCG and a placebo. Consuming 135 mg of EGCG decreased the frontal cortex’s cerebral blood flow compared to the placebo. However, the study found that both 135 mg and 270 mg doses of EGCG did not significantly change cognitive performance or mood [15].

EGCG has potential therapeutic benefits for NDs. It targets protein misfolding and aggregation. It also interacts with misfolded proteins like α-synuclein and amyloid-β (Aβ)-peptide. It provides promising drug discovery options for treating these diseases [16]. In addition, EGCG has neuroprotective and neuroinflammatory properties, which have shown promise in reducing AD [17]. Tea Catechins improve cognitive abilities, prevent Aβ plaque formation, and offer antioxidant benefits, potentially aiding dementia and AD treatment [18]. Green tea can help protect the aging brain and reduce the incidence of dementia, AD, and PD [19]. TSCI can lead to motor paralysis, sensory anesthesia, and autonomic dysfunction, with some patients also experiencing neuropathic pain. The primary pathological alterations in SCI include elevated potassium and glutamate levels, cell necrosis, disruption of spinal cord pathways, and a breached blood brain barrier (BBB) [20]. Wistar rats were given 50 mg/kg EGCG or 0.9% saline daily for two days after hypoxia–ischemia (HI) induction and one day and a half before. After exposure to an 8% oxygen/92% nitrogen atmosphere, EGCG significantly decreased infarct volume and inducible nitric oxide synthase (iNOS) activity. However, it upregulated NOS protein expression in endothelial and neuronal cells, maintaining mitochondrial energetics [21]. EGCG regulates hippocampal and apoptosis-related proteins to prevent neurons from harm. It has a neuroprotective impact by regulating apoptosis and hippocampal expression in ischemia neuronal injury. It also treats ischemic stroke [22]. In addition, EGCG is shown to restore mitochondrial function and cellular damage after subarachnoid hemorrhage (SAH)-induced improvement in autophagic flux. It can control the expressions of Atg5, LC3B, and Becn-1 mRNA, restoring disturbed autophagy flow and reducing cell death [23]. EGCG impacts the MCAO model’s capacity to inhibit apoptosis. It prevents neuronal cell death by inhibiting the apoptotic pathway [24].

Moreover, EGCG therapy improved learning and memory in stressed rats and stopped the decline in locomotor activity [25]. EGCG has neuroprotective effects of EGCG in a transgenic mouse model of ALS. The mice were given EGCG and vehicle-treated control groups, and their motor function was assessed. EGCG therapy significantly delayed disease onset and increased life expectancy. The spinal cords of EGCG-treated mice showed decreased microglial activation, NF-κB reactivity, and protein levels [26]. Furthermore, EGCG may have therapeutic potential as an ALS disease-modifying medication [27]. This review showed that EGCG has significant neuroprotective properties in various NDs. It reduces OS, inhibits Aβ aggregation, and modulates cell signaling pathways. It also has anti-inflammatory properties, promoting mitochondrial function and autophagy. Moreover, it may improve cognitive performance and reduce neurodegenerative biomarkers.

## EGCG: bioavailability and neuroprotective properties

The low bioavailability of EGCG is a critical factor to consider when comparing in vitro results to in vivo tests. The causes behind the inconsistent bioavailability of EGCG are both recognized and unidentified. Pharmacokinetic characteristics can be a significant tool in determining an intervention’s appropriate dosage and frequency. EGCG is primarily absorbed in the small intestine and transported to the large intestine, further broken down by microbiota, preventing its entry into the bloodstream [28–30]. A healthy individual can reach peak plasma concentrations of EGCG in 1–2 h after a single oral dosage in the morning after an overnight fast. In a whole day, these levels progressively drop to undetectable levels. EGCG has an elimination half-life of 3.4 ± 0.3 h [28]. A study found the bioavailability of EGCG in the brain. Rats receiving a high oral dose of 500 mg/kg body weight showed plasma and brain EGCG concentrations of 12.3 nmol/mL and 0.5 nmol/g, respectively [31]. The brain’s radioactivity was assessed six hours after the initial treatment and increased significantly after a second administration. EGCG builds up in the brain after repeated administration [32]. Six human volunteers consumed 250 ml of green tea, but HPLC–MS analysis demonstrated flavan-3-ol methyl-glucuronide and sulfate metabolites entered the bloodstream but did not cross the blood-cerebrospinal fluid barrier [33]. The differences between data from animals and humans may be due to three things: (1) the very high-dose of EGCG used in the animal model compared to the single, relatively small dose found in one cup of tea; (2) the clinical study’s choice of time point for the lumbar puncture (2 h after drinking); and (3) an apparent faster rate of catechin metabolism in humans. According to in vivo microdialysis of the rat hippocampal region conducted after intravenous administration of these fundamental flavanol monomer units, catechins and epicatechins cross the BBB [34].

The application of EGCG in humans has been limited due to poor bioavailability, membrane permeability, and stability. Innovative approaches, such as structural alterations, nanocarriers, and cooperation with other bioactivities, are needed to improve EGCG’s chemical-biological characteristics and therapeutic potential [35]. Another study found that cotreatment with piperine significantly enhanced the bioavailability of EGCG in mice [36]. EGCG is effective against various NDs but has limited pharmacological efficacy due to chemical instability and low bioavailability. It increased bioavailability and protection against degradation, making it a potential substitute delivery method for oral EGCG [37]. Additionally, EGCG has neuroprotective and neuroinflammatory properties, which have shown promise in reducing AD. It proposes novel preventive strategies for treating these diseases [17]. EGCG is a free radical scavenger and has been demonstrated in animal studies to possess anti-aging properties [38]. Moreover, EGCG influences a wide range of possible AD targets. It prevents Aβ-induced neurotoxicity in cultured hippocampus neurons. It has been shown to convert APP into sAPP by activating protein kinase C, thus inhibiting the synthesis of neurotoxic Aβ [39]. EGCG inhibited Aβ neurotoxicity by stimulating GSK3 and restricting cAbl/FE65 [40]. Furthermore, EGCG protects neurons by observing its biological, pharmacological, antioxidative, and metal-chelating properties. It can activate different brain cellular processes linked to AD and PD [41]. However, EGCG has potential neuroprotective effects, improving neurogenesis, modulating inflammation, and reducing OS. Despite its low bioavailability, it prevents NDs.

## Epigallocatechin 3-gallate in neurodegenerative diseases

EGCG can slow the progression of NDs by reducing OS, reducing neuroinflammation, and controlling protein misfolding and aggregation. It is a promising treatment for these diseases (Table 1).

**Table 1:** EGCG prevents and treats NDs through various mechanisms

Disease name	Study model	Dose/conc	Findings	Reference
Alzheimer’s disease	Male Sprague–Dawley rats	100, 250, and 625 mg/kg	Protected rats from AD-induced learning and memory impairments	[42]
	SAMP8 mice	15 mg/kg	Improved memory and restored abnormal synaptic protein levels in AD mice’s frontal cortex and hippocampus	[43]
	Male Wistar rats	10 mg/kg	Protected against Aβ-induced memory and coordination impairment in rats	[44]
	APPswe/PS1dE9 mice	40 mg/kg	Reduced Aβ plaque burden and improves synaptogenesis, memory, and learning, potentially reducing cognitive impairment in AD	[45]
	Transgenic mice	50 mg/kg	Reduced cognitive impairment and modulate tau pathology in AD	[46]
	APP/PS1 mice	50 mg/kg	Decreased Aβ and BACE1 protein expression and improved synaptogenesis, memory, and learning	[47]
	Tg APPsw transgenic mice	–	Reduced Aβ generation in mice and murine neuron-like cells transfected with the Swedish mutant amyloid precursor protein	[48]
	Mice	1.5 and 3 mg/kg	Prevented memory impairment and amyloidogenesis by inhibiting neuroinflammatory-related cytokines released from astrocytes	[49]
	Male Wistar rats	10 mg/kg	Reversed OS and decreased acetylcholinesterase activity in a streptozotocin-induced dementia model	[50]
	APP/PS1 transgenic mice	50 mg/kg	Reduced cognitive impairment in APP/PS1 mice, improved dendritic integrity, and reduced Aβ plaques	[51]
	APP/PS1 mice	30 mg/kg	Enhanced cognition and decreased in AD mice	[52]
	Mice	5 and 15 mg/kg	Reduced cognitive decline in AD model mice	[53]
Parkinson’s disease	Male C57 black mice	25 mg/kg	Regulated ferroportin in SN, reduces OS and aids in neuroprotection against MPTP-induced functional and neurochemical impairments in mice	[54]
	C57BL/6 J mice	25 and 50 mg/kg	Prevented MPTP toxicity, recovered movement, increased T cell ratio, and decreased inflammatory markers	[55]
	Male Sprague–Dawley rats	50 mg/kg	Improved neurobehavioral symptoms in PD rats by activating the AKT/GSK-3β/mTOR pathway	[56]
	Male Sprague–Dawley rats	10 mg/kg	Treated LPS-induced neurotoxicity by decreasing inflammatory mediators and maintaining DA levels in the midbrain	[57]
	Male C57B6 mice	25 mg/kg	Prevented MPTP-induced reductions in dopaminergic neurons, TH activity, and striatum in mice	[58]
	Male C57Bl/6 mice	10 and 50 mg/kg	Reduced neuronal death rate in MPTP mice and iNOS expression	[59]
	Mice	2 and 10 mg/kg	Protected dopamine neuron loss and depletion in striatal dopamine and tyrosine hydroxylase protein levels	[60]
	Male Wistar rats	100 or 300 mg/kg	Prevented most ROT-induced motor impairments, reduced nitric oxide levels, and increased enzyme activity	[61]
	Female C57BL/6 N mice	10 mg/kg	Decreased anxiety-like behavior and motor impairments, improved PFF-induced degeneration of TH immuno-positive neurons	[62]
Multiple sclerosis	Male C57BL/6 mice	50 mg/kg	Increased Plp and Olig1 expression in the cerebral cortex of cuprizone-induced MS mice	[63]
	C57BL/6 mice	50 mg/kg	Enhanced the expression of PLP and Olig1 in the cerebral cortex of a mouse model of MS induced by cuprizone	[64]
	Male C57BL/6 mice	22.5 mg/kg	Decreased EAE severity and inflammation in the CNS, and suppressed M1 macrophage-mediated inflammation	[65]
	–	800 mg	Decreased body fat, improved inflammation, and oxidation in MS	[66]
	Female C57BL/6 mice	–	Decreased clinical symptoms and pathology of EAE in mice by regulating the balance of CD4 + T cell subsets	[67]
Spinal cord injury	Male Wistar rats	50 mg/kg	Improved neuroregeneration after SCI by regulating inflammatory cytokine levels	[68]
	Female Sprague–Dawley rats	20 mg/kg	Improved locomotor recovery, sensory neurobehavior, neuron number, and reduced lesion area in SCI	[69]
	Female Balb-c mice	–	Decreased thermal hyperalgesia and gliosis through the FASN and RhoA pathways, thereby decreasing cytokines in the spinal cord	[70]
	Female Sprague–Dawley rats	10 or 20 mg/kg	Improved functional recovery after SCI by enhancing the expression of BDNF and GDNF	[71]
	Male Wistar rats	50 mg/kg	Decreased MDA levels, reduced caspase-3, TNF-α, and iNOS expression, and reduced spinal cord neuron degeneration	[72]
	Male Sprague–Dawley rats	50 mg/kg	Prevented the rat spinal cord from secondary damage by regulating inflammatory reactions	[73]
	Male Wistar rats	17 mg/kg	Enhanced axonal sprouting, decreased glial scar formation, and altered cytokine levels	[74]
	Female Sprague–Dawley rats	50 mg/kg	Protected spinal cords from secondary injuries	[75]
	Male Sprague–Dawley rats	100 mg/kg	Reduced edema and astrocytic swelling in rats after SCI by down-regulating AQP4 expression and GFAP expression	[76]
	Balb/c mice	–	Decreased thermal hyperalgesia in mice with chronic constriction injury of the sciatic nerve	[77]
	Male Wistar rats	50 mg/kg	Suppressed mTOR signaling pathways, resulting in recovery from SCI in rats	[78]
	Male Wistar rats	50 mg/kg	Improved spinal cord neuronal degeneration	[79]
Brain injury	Male Sprague–Dawley rats	100 mg/kg	Reduced the risk of TBI by inhibiting the formation of edema and OS	[80]
	Male Wistar rats	–	Enhanced the number of neural stem cells in the damaged area after a TBI in rats	[81]
	Rats	10 and 20 mg/kg	Prevented brain damage caused by homocysteine in rats	[82]
	Male Sprague–Dawley rats	20 mg/kg	Decreased neuronal apoptosis in rats after middle cerebral artery occlusion injury	[83]
	Male Sprague–Dawley rats	50 mg/kg	Decreased inflammation and prevented focal cerebral ischemia/reperfusion injury	[84]
	Male rats	50 mg/kg	Reduced neuronal cell damage in rats induced by focal cerebral ischemia	[85]
	Male Sprague–Dawley rats	50 mg/kg	Prevented brain damage in rats after transient middle cerebral artery occlusion	[86]
	Male Sprague–Dawley rats	50 mg/kg	Improved iron accumulation, apoptosis, and neuronal regeneration in the hippocampus, and promoted memory	[87]
Stroke	Male C57BL/6 mice	50 mg/kg	Improved angiogenesis in a mouse of ischemic stroke by enhancing the Nfr2 signaling pathway	[88]
	Male Sprague–Dawley rats	–	Inhibited endoplasmic reticulum stress in a stroke model in rats	[89]
	Sprague–Dawley rats	50 mg/kg	Improved neuronal damage in ischemic stroke	
	Male C57BL/6 mice	–	Improved neurogenesis in adult mice after an ischemic stroke	[90]
Others	Female Balb-c mice	50 mg/kg	Reduced neuropathic pain in mice after chronic constriction nerve injury	[91]
	Male Wistar rats	50 mg/kg	Reduced peripheral nerve degeneration in rats suffering from sciatic nerve injury	[92]

### Alzheimer’s disease

**Fig. 1:**
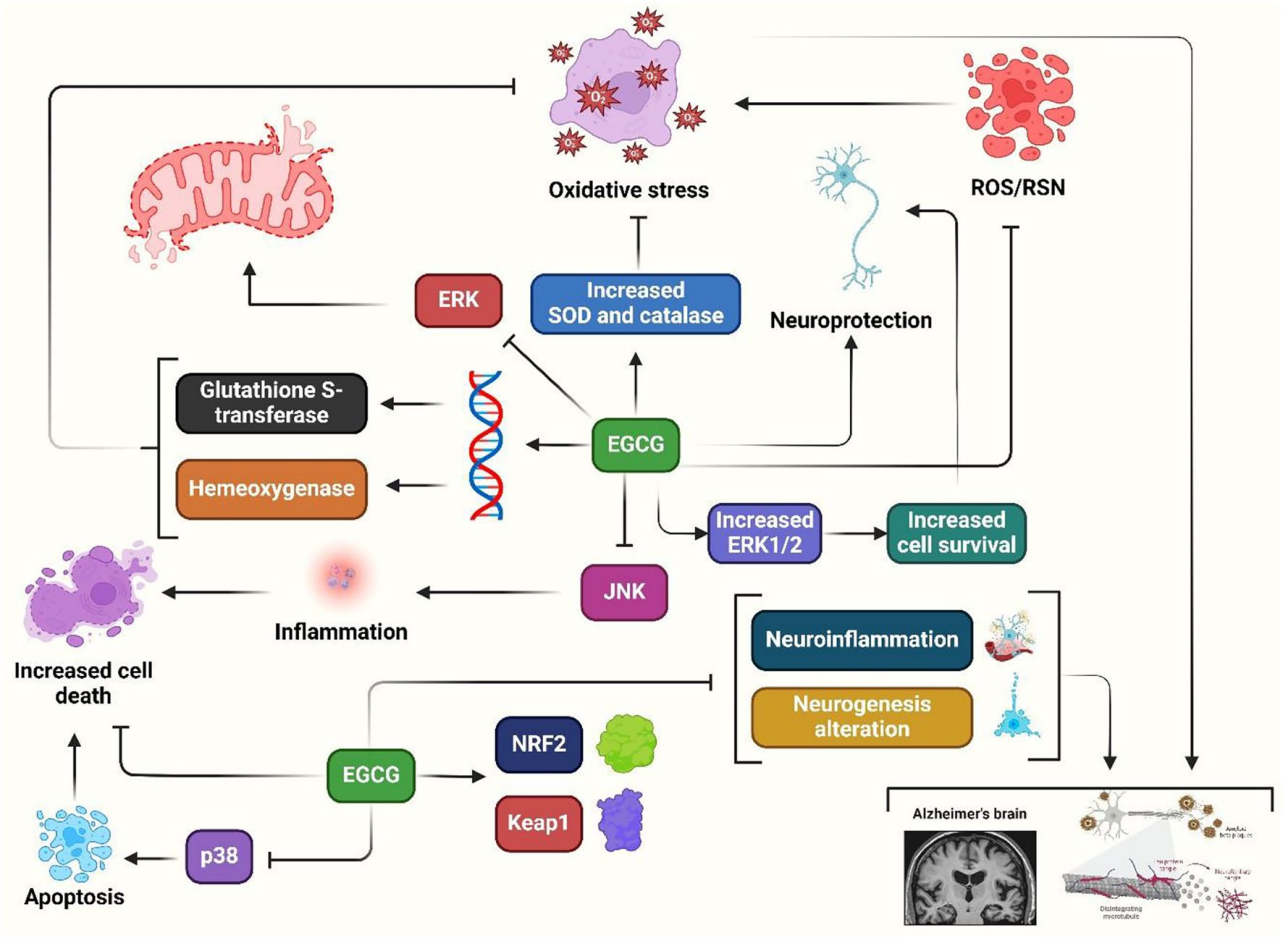
EGCG inhibits OS by promoting stress response genes, increasing catalase and SOD activities, and exhibiting neuroprotective effects in AD

AD is an ND that impairs cognition and memory, with OS playing a role in its onset and progression. Persistent viral infections like HCV, HHV-1, CMV, and HHV-2 may cause neuropathology. EGCG prevents and treats AD (Fig. 1) [93]. EGCG regulates inflammatory processes, stimulates neuronal survival pathways, and reduces tau hyperphosphorylation and aggregation [94]. Additionally, EGCG showed promising results in mouse models of AD and HIV-associated dementia. However, clinical use has been challenging due to poor bioavailability and ineffective delivery [95]. Preclinical research suggests EGCG in tea may have neuroprotective and preventative effects against AD [96]. A study found that EGCG administration can help rats with Aβ-induced cognitive impairment by improving coordination and memory. The study involved four groups of male Wistar rats: lesion, sham-operated, sham-operated, and EGCG-pretreated lesion. The lesion group showed lower psychomotor coordination and spontaneous alternation behavior compared to the sham group, similar to AD behavioral alterations. However, the EGCG pretreatment group showed improvements in these areas. EGCG can potentially help rats with Aβ-induced cognitive impairment [44]. EGCG/AA nanoparticles showed improved stability and increased therapeutic concentrations in the brain. Oral administration of EGCG/AA NPs to APP/PS1 mice led to increased synaptophysin expression, decreased neuroinflammation, reduced amyloid plaque burden, and improved spatial learning and memory. The mechanism suggests that increased therapeutic concentrations in the brain are due to EGCG stabilization in NP complexes and a destabilized BBB [45]. Another study found that long-term oral administration of EGCG enhanced memory function decreased tau hyperphosphorylation and Aβ levels, and corrected lowered levels of postsynaptic density protein 95 and synaptophysin in a senescence-accelerated mouse model [43]. AD is a result of mitochondrial dysfunction caused by Aβ. In vitro studies showed luteolin and EGCG as top mitochondrial restorative substances. In vivo testing on an AβPP/PS-1 mouse model showed that EGCG administration increased mitochondrial parameters by 50–85% [97].

EGCG decreased cellular holo-APP without changing APP mRNA quantities and inhibited the translation of a reporter gene for luciferase. Fe_2_SO_4_ counteracted EGCG’s effects, suggesting it may be a viable treatment for AD and other iron-related diseases [98]. Oral EGCG administration in drinking water also reduced Aβ deposition. Both oral and intraperitoneal administration of EGCG showed cognitive benefits, with the effects of i.p. treatment being more noticeable [46]. Combining EGCG and a therapeutic gene in a multifunctional nanocarrier significantly increased spatial learning and memory capacities in APP/PS1 and wild-type mice, suggesting that co-delivering these molecules may be highly effective in AD treatment [47]. A study investigated the impact of EGCG on cholinergic-like neurons (ChLNs) with the PRESENILIN 1 mutation E280A (PSEN1 E280A). EGCG significantly inhibited transcription factors, blocked p-TAU, and reduced aggregation of sAPPβf in mutant ChLNs. It also inhibited NF-κB activation, reversed Ca^2+^ influx dysregulation, and reduced pro-inflammatory IL-6 secretion in wild-type astrocyte-like cells [99]. BACE-1, an enzyme that limits Aβ synthesis, is upregulated in neural cells due to OS. Curcumin and EGCG inhibit BACE-1 increase, reducing the generation of ROS and β-sheet structure [100]. Another study found the effects of sub-chronic EGCG therapy on memory and cognition in rats with AD. The rats were given a daily dose of 10 mg/kg of EGCG for four weeks, and Morris’ Water Maze was used to assess memory and learning. EGCG administration completely reversed cognitive impairments and S100B content [50]. Moreover, a study investigated the potential benefits of EGCG against memory impairment, amyloidogenesis, and neuroinflammation caused by systemic inflammation. It prevented memory loss and apoptotic neuronal cell death in mice after intraperitoneal injections of lipopolysaccharide. It also inhibited the rise in Aβ-peptide levels, astrocyte activation, and inflammatory proteins involved in astrocyte activation and amyloidogenesis [49]. EGCG decreased cognitive impairments in APP/PS1 mice, improved dendritic integrity, and reduced Aβ plaques [51].

Coenzyme Q10 and EGCG have positive effects on the CNS. A study in rats found that protein-malnourished rats had more pronounced brain neurological damage, indicative of AD induction. When EGCG and CoQ10 were administered during AD production, both NF and PM rats exhibited protective effects, with decreased Aβ, acetylcholinesterase (ACHE), and MDA and elevated SOD and TAC. Combining EGCG and CoQ10 therapy significantly protects against AD induction in both NF and PM mice [101]. EGCG reduced the generation of AD in mice. It increased the cleavage of the APP’s *α*-C-terminal fragment, leading to increased hydrolysis of α-secretase. It also reduced Aβ levels and plaques in mice overproducing Aβ [48]. A study found the inhibitory mechanisms of genistein and EGCG on the conformational alterations of Aβ42 peptides. Genistein and EGCG suppress the Aβ42 peptide’s conformational transition and decrease its *β*-sheet secondary structures ratio [102]. Another study showed that intragastric delivery of low and high-dose EGCG over 60 days can prevent Aβ buildup and cognitive decline in senescence-accelerated mice P8. It suggested a correlation between increased NEP expression and EGCG-induced Aβ decrease. EGCG can effectively prevent the downregulation of Aβ [53]. In addition, a study investigated the neuroprotective impact of vinpocetine in rat models of aluminum chloride-induced AD. The combination of vinpocetine and EGCG showed the strongest neuroprotection, with a decline in Aβ and ACHE levels [103]. Moreover, EGCG decreased Aβ-induced memory impairment in mice. It reduced brain α-secretase activity, increased brain *β*- and *γ*-secretase activity, and inhibited the production of metabolic products from APP, C99, and Aβ. It also suppressed nuclear transcription factor-κB and prevented apoptotic neuronal cell death [104]. A study involving transgenic mice showed that combined EGCG and FA therapy corrected cognitive impairment at 15 months. The combined therapy improved brain parenchymal and cerebral vascular Aβ deposits and reduced Aβ-proteins [52]. The Aβ peptide is linked to AD, with nitrosative and OS playing a role in its damage. EGCG reduces apoptosis, nitrosative stress, and nitric oxide formation in BV2 microglia [105]. Furthermore, EGCG significantly reduced the production of proinflammatory cytokines and neurotoxins in microglia. It restored intracellular antioxidants, preventing ROS from inducing NF-κB activation. It also prevented Aβ-mediated cytotoxicity of neuro-2a neuronal cells, suggesting it could be a useful drug to stop inflammatory neurodegeneration caused by Aβ [106].

### Parkinson’s disease

**Fig. 2:**
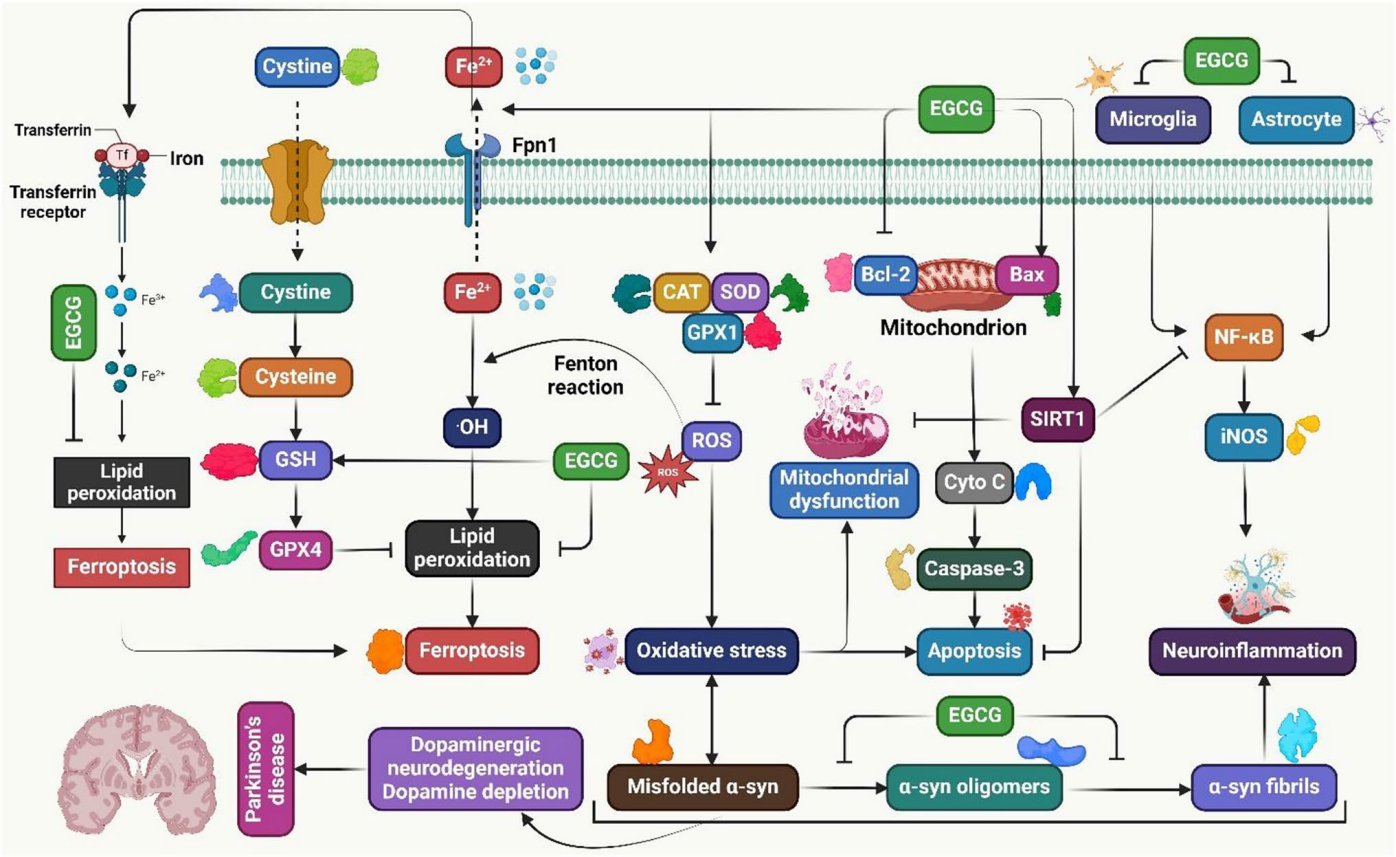
EGCG exhibits neuroprotective benefits in PD, inhibiting α-synuclein aggregation, oligomerization, fibrillation, OS, neuronal death, protein misfolding, and neuroinflammatory reactions

PD is an ND characterized by the loss of dopamine and neuronal degeneration in the SN pars compacta. Anti-oxidant and anti-inflammatory medications are suggested to slow PD progression. EGCG has strong neuroprotective benefits against neuronal cell death, OS, neuroinflammation, and protein aggregation (Fig. 2) [107]. EGCG may prevent PD by acting as a neurorescue against MPTP-induced impairments in mice and decreasing OS [54]. A study showed the potential of EGCG in PD treatment by analyzing its impact on the peripheral immune system. EGCG administration prevents MPTP toxicity, recovers movement behavior, and decreases inflammatory markers [55]. Another study showed significant differences in the EGCG group’s rotation speed, left forelimb utilization, neuron apoptosis, *α*-synuclein expression, mTOR, AKT, and GSK-3β protein expressions, and neuron apoptosis [56]. EGCG prevents the aggregation of SNCA. It can remodel SNCA aggregates and is a promising medication for PD and other α-synucleinopathies [108]. A study found the potential of EGCG as an antioxidant to mitigate OS damage in PD. It prevented OS and NLCs from rotenone, decreased cell death markers, and preserved mitochondrial membrane potential [109]. Additionally, another study showed that natural antioxidants significantly inhibit OS-induced apoptosis in catecholaminergic PC12 cells. EGCG significantly prevents 6-OHDA-induced cell death [110]. EGCG prevents dopaminergic neurons from lipopolysaccharide-induced neurotoxicity and prevents the production of inflammatory mediators. EGCG may have therapeutic benefits by lowering inflammatory mediators [57]. NO is a key mediator of MPTP’s toxicity in PD. Blocking NO synthase activity in the brain can counteract MPTP-induced PD. EGCG can reduce MPTP-induced PD by suppressing NOS expression in the substantia nigra. The suppressive effects of EGCG may be due to NO suppression [58]. Mice were given EGCG at different doses, and the lower doses reduced neuronal death. It may inhibit iNOS, potentially preventing MPTP toxicity [59].

EGCG inhibited SOD and catalase activity in the striatal membrane [60]. A study using PQ-injured PC12 cells as an in vitro model of PD showed that EGCG reduced PQ-induced apoptosis, possibly by preserving mitochondrial membrane potential, suppressing caspase-3, and reducing Smac production [111]. Studies on dopaminergic neuroblastoma SHSY-5Y cells showed that EGCG decreased cell death induced by DDT. EGCG can prevent organochlorine pesticide-induced cell damage, with the preventive effect positively linked to the quantity of exposures [112]. EGCG treatment effectively prevents motor deficits, reduces lipid peroxidation, and reduces apoptotic and neuroinflammatory markers [61]. Another study found the effectiveness of EGCG in reducing brain damage caused by α-synuclein fibrils in a chronic PD mouse model, showing reduced anxiety-like behavior and motor impairments after six months [62]. EGCG can cross the BBB. EGCG has a protective mechanism in PC12 cells that may provide new insights into PD prevention and treatment [113]. Additionally, EGCG can protect neurons from OS in PD. The research used human neuroblastoma SH-SY5Y cells treated with 6-OHDA as a model. EGCG significantly reduced 6-OHDA-induced cell death and promoted neural cell proliferation [114]. Moreover, a study found the potential of EGCG as a drug for PD prevention and treatment, showing its ability to prevent amyloid fibrillation and inhibit *α*-synuclein-induced cell death [115]. EGCG prevents inflammation and protects neurons. EGCG-loaded liposomes showed anti-inflammatory effects against BV-2 microglial cells and reduced pro-inflammatory cytokines in the Sprague Dawley rats’ SN. EGCG-loaded liposomes may be a promising treatment for PD [116]. A study showed that EGCG extended life span and motor activity in wild-type Canton-S flies exposed to paraquat (PQ). The study found an animal model of PD that considers environmental neurotoxicants and gene alterations. The flies were treated with EGCG, which prevented and protected against PQ-induced decrease in life expectancy, motor function, lipid peroxidation, and neurodegeneration [117].

Another study found that EGCG has strong neuroprotective effects on PD mice and human neuroblastoma cells. It reduced cell mortality caused by 6-OHDA exposure. It also recovered ERK1/2 and PKC activity from 6-OHDA toxicity, but PKC inhibitor GF 109203X eliminated its neuroprotective effect. Gene expression studies showed that EGCG inhibited mRNA production [118]. EGCG increases spike frequency, reduces resting membrane potential, and reduces afterhyperpolarization amplitude. Dopamine release is closely linked to neural activity. It enhances neuronal activity by blocking calcium-dependent potassium currents and potentially triggering NMDA-dependent action potential bursts, similar to apamin or bicuculline methiodide [119]. Furthermore, a study aimed to determine if pro-drug completely acetylated EGCG (pEGCG) can provide greater neuroprotection in Parkinsonism. The pEGCG can lower lactate dehydrogenase release and decrease caspase-3 activity but not decrease caspase-3 activity caused by 6-OHDA. Western-blot analysis suggested that pEGCG may stimulate Akt signaling pathways for neuroprotection [120]. EGCG has shown potential in PD treatment and prevention due to its antioxidant, anti-inflammatory, and anti-apoptotic properties. It reduces α-synuclein aggregates, enhances dopamine production, and preserves mitochondrial health.

### Multiple sclerosis

MS is an autoimmune disease that impacts the CNS. EAE showed neuronal disease is the major cause of clinical impairment. Combining Glatiramer acetate (GA) with EGCG showed synergistic protective effects against neuronal cell death and regeneration. EGCG could be an adjuvant therapy for neuroinflammatory and NDs, highlighting the benefits of combining neuroprotective and anti-inflammatory therapies [121]. EGCG can enhance energy metabolism in MS patients. Women receiving EGCG had higher postprandial energy expenditure and CHOx levels than men. EGCG affects autonomic and endocrine regulation and may be responsible for these differences [122]. A study found the safety and effectiveness of adding EGCG to GA in patients with relapsing–remitting MS. EGCG added to GA did not improve safety or effectiveness over 18 months, and up to 800 mg daily was [123]. EGCG upregulates the expression of Olig1 and PLP in the cerebral cortex of a mouse model of cuprizone-induced demyelination [63]. Additionally, EGCG and coconut oil significantly reduced anxiety, functional impairment, and IL-6 levels in MS patients, thereby enhancing their functioning abilities. The reduction in IL-6 may be due to the Mediterranean diet’s antioxidant potential and ability to raise BMI [124]. EGCG can suppress EAE by lowering neuronal damage and brain inflammation. Oral treatment reduced proliferation and TNF-α generation in mice, and EGCG prevented TRAIL-induced neuronal injury in living brain tissue [125]. Another study showed the potential benefits of EGCG in autoimmune diseases. It reduces autoimmune responses by adjusting the ratio of pro- to anti-autoimmune CD4 + T-cell subsets [126]. EGCG-treated mice had significantly higher PLP and Olig1 expression than the SHAM and control groups. This suggests increased PLP expression may be involved in the remyelination process, as PLP is a key component of the myelin sheath [64].

EGCG and GA can be beneficial for mice with chronic EAE. EGCG downregulated heme oxygenase-1 (HO-1) gene expression in afflicted CNS areas, but GA + EGCG combined therapy increased HO-1 expression. EGCG-induced neuroprotection in chronic EAE can involve controlling oxidative mechanisms, such as HO-1 downmodulation [127]. A study found that EGCG, an immunomodulatory drug, reduced the expression of the RORC2 gene in MS patients’ peripheral blood mononuclear cells [128]. EGCG may effectively treat MS by reducing inflammation and inflammation in the CNS. It induced M2 macrophage polarization while inhibiting M1 macrophage polarization, possibly due to its suppressing NF-κB signaling and glycolysis in macrophages [65]. In addition, a study evaluated the impact of a dietary intervention with coconut oil and EGCG on gait and balance. 51 MS patients were divided into two groups: one receiving EGCG and coconut oil daily, and the other a Mediterranean diet. Results showed significant improvements in quadriceps muscle strength and quantitative balance [129]. Moreover, another study showed the impact of EGCG on retinal thickness analysis in MS patients. Results show no long-term effects on pRNFL, GCIP, or INL, suggesting insufficient evidence to support EGCG’s neuroprotective effects on retinal thickness in individuals with PPMS and SPMS. The findings are consistent with the primary SUPREMES trial but may lack sufficient power to identify a treatment effect [130]. EGCG can treat dyslipidemia. IG patients had reduced triglycerides (TG) compared to CG patients, with a positive correlation between EDSS and C-reactive protein (CRP). The decrease in body fat may be linked to these IG alterations. The functional gains may be explained by a drop in blood TG levels observed after EGCG and coconut oil treatment [131]. A study found the effects of coconut oil and EGCG on cortisol activity in MS patients. The intervention group showed a significant increase in albumin, possibly due to a drop in serum-free cortisol. Additionally, the intervention group experienced a decline in belly fat and sadness scores. Coconut oil and EGCG can reduce depression in MS patients [132].

EGCG has been shown to have an immunomodulating effect on the immune system, particularly T cell functions. Studies using animal models of autoimmune disorders show improvements in conditions treated with EGCG. EGCG is used to prevent and treat T-cell-mediated autoimmune disorders [133]. Another study investigated the impact of EGCG on autoimmunity in humans with MS. It suppressed transcription factors for Th1 and Th17 differentiation [67]. Moreover, a study showed the role of haptoglobin. This protein is linked to the development of MS as a potential marker of muscle improvement after EGCG administration and increased beta-hydroxybutyrate levels [134]. Furthermore, EGCG modified CD4 + T cell subpopulations and reduced autoimmune reactions. It inhibited the formation of Th1, Th9, and Th17, as well as Treg development caused by IL-6. It also suppressed transcription factors. It influenced CD4 + T cell lineage differentiation by affecting their regulatory networks, leading to reduced autoimmune reactions [135].

### Spinal cord injury

SCI can cause autonomic dysreflexia, chronic discomfort, and permanent motor and sensory impairments. A study tested the effectiveness of EGCG intravenous infusion for 36 h in rats with acute and chronic SCI. The study demonstrated improved motor and sensory functions, BBB, tactile allodynia, and mechanical nociception in rats with SCI [69]. Another study showed the neuroprotective effects of EGCG on SCI healing in male Wistar rats. EGCG treatment improved behavioral performance, increased axonal sprouting, and favorable glial scar remodeling. Cytokine levels were measured after SCI, showing a decrease in inflammatory cytokine levels. EGCG reduced nuclear translocation of the NF-κB dimer’s subunit p65, attenuating the canonical NF-κB pathway. It has therapeutic benefits for SCI, including increased axonal sprouting and improved behavioral function [68]. Additionally, a study evaluated the effects of EGCG administration on thermal hyperalgesia, spinal cord gliosis, and cytokine expression in mice following spinal cord contusion. EGCG therapy reduced thermal hyperalgesia and gliosis in mice but did not affect locomotor recovery [70]. Another study investigated the therapeutic benefits of subarachnoid injections of EGCG in rats with SCI. Rats treated with EGCG showed improved locomotor function, reduced myelin loss, increased Bcl-2 expression, and decreased Bax expression [71]. Moreover, a study found that subarachnoid injections of EGCG in rats with SCI improved locomotor function, reduced myelin loss, increased Bcl-2 expression, and decreased Bax expression [71]. A study investigated the neuroprotective benefits of EGCG on the brain after spinal cord ischemia–reperfusion injuries (IRI). It may protect the spinal cord against IRI by reducing MDA levels, reducing caspase-3, TNF-α, and iNOS expression, and reducing neuron degeneration [72]. EGCG treatment groups had significantly lower MPO activity, reduced production of these inflammatory responses, and reduced myelin deterioration [73].

In addition, a study found the effects of combining curcumin and EGCG therapy on SCI in rats showed considerable behavioral improvement. Curcumin and EGCG therapy combined improved axonal sprouting, reduced glial scar formation, and altered cytokine levels, but their synergistic effect was less noticeable. Although not as effective as monotherapy, the combination of curcumin and EGCG therapy resulted in behavioral recovery for patients with experimental SCI [74]. EGCG treatment groups had lower MDA levels, increased Bcl-2 expression, reduced Bax expression, lower TUNEL-positive rate, decreased lesion area, and enhanced behavioral function [75]. Moreover, EGCG administration can reduce neuropathic pain in mice with chronic constriction injuries. Balb/c mice were given EGCG or a vehicle for the first week after the procedure. The therapy reduced thermal hyperalgesia in mice with CCI injuries by down-regulating CX3CL1 protein expression in the spinal cord [77]. A study on male adult Wistar rats found that EGCG inhibits OS and promotes sciatic nerve regeneration after crush injuries. Treatment improved foot position, motor recovery, early jumping, reduced nociception deficits, and accelerated total antioxidant capacity healing [136]. Another study investigated the neuroprotective properties of EGCG after sciatic nerve transection. After nerve transection, EGCG-treated rats showed higher SOD and CAT activity, lower MDA levels, and reduced caspase-3 and COX-2 production. They also showed a reduction in S100B expression. EGCG may prolong neuron survival and protect against retrograde apoptosis, potentially preventing neuronal damage [137]. EGCG significantly reduced neurological damage, including memory and spatial learning, and reduced oxidative damage and inflammation. It increased AMPK phosphorylation [138]. Additionally, a study showed the effects of EGCG and hyperbaric oxygen on rats recovering from SCI and found that combining both treatments significantly improved neurological functions, IL-10 gene expression, and antioxidant factors [139]. However, EGCG administration reduced the expression of NGFR-p75 on days 1 and 3 following nerve damage. It has neuroprotective action on spinal cord neurons that may be mediated through neurotrophic factor regulation [140]. Furthermore, EGCG can reduce stress. Combining high-potency medication loading with exosomes for spinal cord regeneration could enhance spinal cord protection [141].

### Brain injury

TBI is caused by mortality and disability. A study on EGCG’s neuroprotective effects on TBI in mice found that it significantly reduced neurological damage, including memory and spatial learning, oxidative damage, and inflammation. It increased AMPK phosphorylation. It also may be a promising therapeutic intervention for TBI-induced TBI [138]. A study found that feeding rats EGCG before or after TBI prevented the production of free radicals, preventing neuronal degeneration and apoptotic death. EGCG administration affected brain morphology and function at different points before and after TBI. It improves cognitive impairment after TBI [142]. EGCG therapy significantly decreased brain water content and vascular permeability, reduced TBI-induced mRNA expression, and prevented microglia activation. It reduced OS, MDA, and NADPH oxidase activation [80]. The EGCG treatment group showed a significant improvement in cerebral functioning and a rise in surviving neuronal cells. It could help TBI sufferers recover [143]. Additionally, EGCG boosts NSC proliferation in brain-injured areas, increasing nestin-positive cells post-TBI but decreasing ssDNA-positive cells and peroxidation. EGCG-containing water can prevent free radical-induced NSC degradation, potentially promoting NSC differentiation into neurons and glia [81]. Another study found that EGCG can reverse homocysteine-induced neurodegeneration and neuroinflammation in hyper-homocysteinemic rats. It reduced apoptotic neurons and neurodegeneration [82]. EGCG reduces neurological function, prevents nerve cells, prevents neuronal death, and reduces OS damage. However, the protective effect diminished after PI3K inhibitors were administered. It may protect against MCAO-induced brain damage [83].

**Fig. 3:**
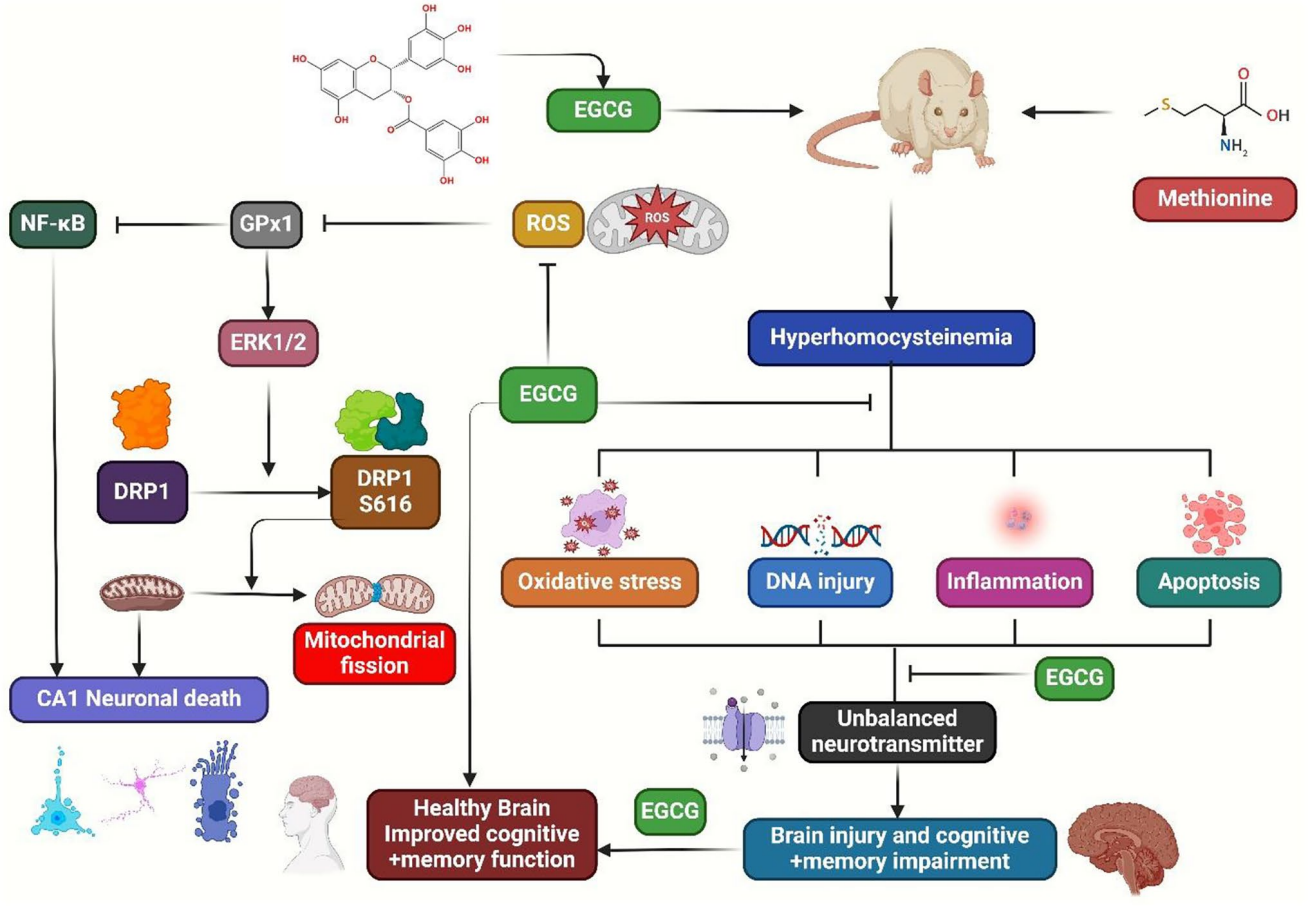
EGCG prevented OS, inflammation, DNA damage, and apoptosis in rats, potentially decreasing cognitive function and memory impairment

Moreover, EGCG has been shown to possess neuroprotective potentials, but its instability could decrease its efficacy. This study develops and improves EGCG-loaded Proliposomes (EGCG-PLs) and investigates their protective impact against TBI in rats. The optimized EGCG-PLs formula demonstrated prolonged in vitro release, increased antioxidant activity, and improved histological changes in brain tissues caused by TBI. EGCG is more effective when prepared in PLs [144]. EGCG reduces inflammation-related chemicals and infarct volume and might be a potential treatment for cerebral IRI [84]. A study investigated EGCG against cerebral ischemia in mature male rats. The rats were given EGCG or a vehicle before MCAO induction, and tests were conducted 24 h after. It improved neurological abnormalities caused by MCAO. It also regulates the apoptotic signaling pathway, acting as a powerful neuroprotective agent during cerebral ischemia [85]. EGCG increased Nissl-positive cells and reduced senescence, inflammation, and brain regeneration. It facilitated brain regeneration in rats treated with acrylamide. It also could mitigate nerve damage caused by acrylamide [145]. Furthermore, another study found EGCG’s potential to reduce brain injury in a rat model, showing its anti-oxidant properties post-reperfusion (Fig. 3) [86]. EGCG protects against cognitive impairment and brain damage caused by methionine. Male mice with hyperhomocysteinemia were given drinking water laced with methionine, and EGCG was given daily. The treatment significantly ameliorated cognitive and memory impairment, improved glutamate and gamma-aminobutyric acid levels, preserved glutathione levels, and antioxidant enzyme activity. EGCG protects against DNA damage and reduces neuroinflammation, decreasing OS and inflammatory cytokines [146]. A study showed that EGCG protected rats from OS, mitochondrial dysfunction, and striatal damage caused by 3-NP. It interacted with nitric oxide modulators. It also reduced 3-NP-induced neurotoxicity, possibly due to nitric oxide regulation [147]. Moreover, another study found the protective benefits and therapeutic mechanisms of EGCG in rats exposed to high-altitude hypoxia. Results showed improved learning and memory abilities, reduced iron buildup, impaired iron metabolism, decreased BDNF, and increased MDA and Caspase-3 expression. EGCG promoted neuronal regeneration and decreased HAH-induced apoptosis, iron accumulation, OS, and cognitive impairment [87].

In addition, a study investigated EGCG on memory in rats suffering from cerebral ischemia. It reduced memory impairment and increased malondialdehyde levels, glutathione, and SOD activity in the brain. It also showed anti-inflammatory properties in microglia cells, reducing neuroinflammation and OS and potentially preventing learning and memory impairments [148]. EGCG protects against corticosterone-induced neuron damage by regenerating the PI3K/AKT and ERK1/2 pathways. It reduces corticosterone-induced neuronal damage. It also mitigates ATP generation, and PGC-1α expression decreases while restoring ERK1/2 and PI3K/AKT phosphorylation [149]. Additionally, EGCG has neuroprotective properties and antioxidative action in cases of ischemic brain damage. It was administered to male Sprague–Dawley rats before MCAO surgery, and after 24 h, it mitigated neurological impairments and reduced thioredoxin expression [150]. Furthermore, a study found the impact of EGCG on behavioral, biochemical, and molecular alterations in rats. The results showed poor retention, impaired spatial navigation, increased acetylcholinesterase activity, and decreased brain weight. EGCG significantly reduced these alterations, suggesting that oxidative-nitrosative stress-mediated apoptotic signaling is activated in cognitive abnormalities linked to fetal alcohol spectrum disorders (FASDs). It is useful in preventing cognitive impairment in FASD-affected children [151]. EGCG mitigates OS, enhances mitochondrial function, and promotes neurogenesis. It improves cognitive function, reduces neuronal death, and supports neurogenesis, with potential therapeutic potential for long-term brain injury recovery.

### Stroke

**Fig. 4:**
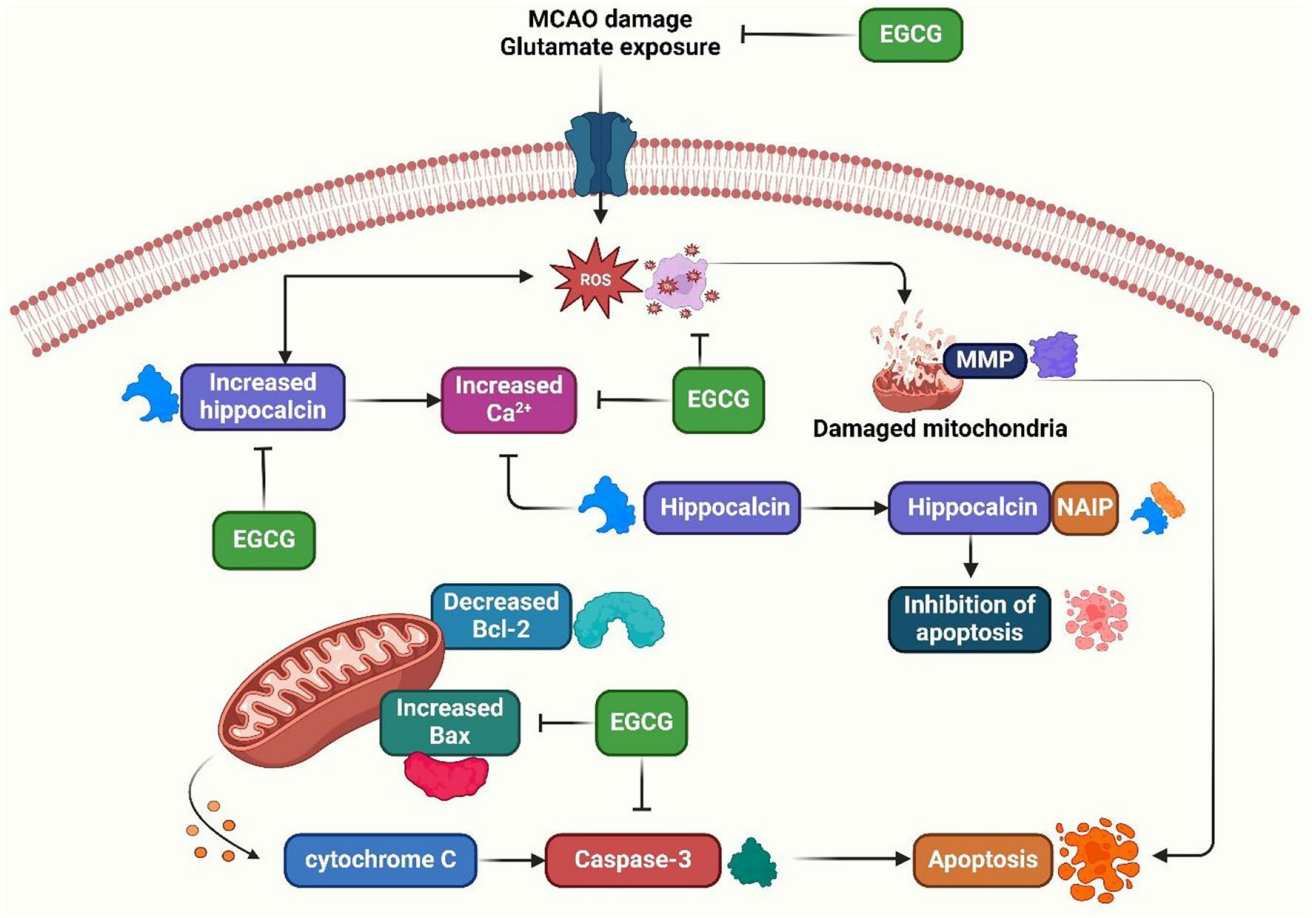
EGCG has a neuroprotective effect on MCAO damage and glutamate toxicity

Stroke is a major global death cause and long-term disability [152]. A study found the impact of EGCG on angiogenesis in mice with temporary MCAO. EGCG administration during an ischemic stroke can stimulate angiogenesis by activating the Nfr2 signaling pathway using a mouse model (Fig. 4). Blocking Nrf2 activation reduced Nrf2 expression, OS, and angiogenic effects in MCAO mice [88]. Another study found the neuroprotective effects of EGCG on rats after acute cerebral ischemia. It reduces endoplasmic reticulum stress indicators, decreases infarct volumes, and increases neurological scores. However, it has a neuroprotective effect that weakens when MEK activity is suppressed [89]. Additionally, a study investigated the long-term effects of EGCG on neurogenesis and functional recovery following ischemic stroke in C57BL/6 mice. EGCG administration increased SVZ NPC proliferation, neuroblast migration, and functional recovery, possibly by inducing the M2 phenotype in microglia. It improved neurogenesis and stroke recovery [90]. A clinical trial evaluating the impact of EGCG on the length of recombinant tissue plasminogen activator therapy window in stroke patients found that patients with delayed onset-to-treatment time had better treatment outcomes after receiving EGCG. This improvement was likely due to decreased plasma levels of MMP-2 and 9 [153]. Moreover, a study evaluated the effectiveness of EGCG in enhancing rat brain function after transient MCAO. Results showed no difference in infarct volume between EGCG-treated and MCAO control groups. EGCG-treated rats showed better forelimb function and normal function [154]. Furthermore, EGCG regulates hippocampal expression in neurons. In a study using ischemic stroke and glutamate-induced neuronal injury, EGCG treatment was found to correct neurobehavioral issues and reduce intracellular calcium excess in glutamate-exposed neurons. It reduced intracellular calcium excess and neuronal cell death. It also regulates caspase-3 and Bcl-2 proteins to protect neurons from glutamate toxicity, thereby reducing the risk of ischemic stroke and brain damage [22]. EGCG reduces OS, supports cell survival, enhances cerebral blood flow, promotes neurogenesis, and protects against long-term deficits.

### Huntington’s disease

HD is characterized by dementia and chorea. Neurotoxic 3-NP impairs animal memory, damaging oxidative defenses and disrupting glutathione levels. The 3-NP model can be used to develop a therapeutic drug for HD. Lycopene and EGCG can improve memory and restore glutathione system functionality despite the reduced glutathione levels [155]. The gene coding region that codes for the protein huntingtin (htt) contains an unstable extension of a CAG repeat. An extended stretch of glutamine is produced by the mutation close to the protein’s NH2 terminus [156, 157]. HD affects 5–10 per 100,000 people in affluent nations due to genetic changes in the htt gene. Treatments for HD symptoms, including behavioral disorders and motor dysfunction, require alternative treatments. EGCG has shown the potential to reduce motor impairments [158]. The neurotoxin 3-NP is a widely used model for studying the pathology of HD. Chronic EGCG administration reduced the memory impairments in rats brought on by 3-NP treatment. 3-NP treatment significantly reduced glutathione levels in rats’ hippocampus, cortical, and stroke regions, reversed by long-term EGCG administration [155]. EGCG effectively suppresses the aggregation of mutant htt exon 1 protein in HD. It modulates the misfolding and oligomerization of mutant htt exon 1 protein in vitro, reducing polyQ-mediated htt protein aggregation and cytotoxicity. This study could lead to the development of an innovative medication for HD and associated polyQ diseases, potentially reducing the toxicity of polyQ [159]. Furthermore, a study evaluated the effectiveness of free and nanoparticle-encapsulated EGCG in treating HD symptoms in mice. EGCG/AA NPs significantly reduced motor abnormalities, depression-like behavior, neuroinflammation, and neuronal death [160].

## Toxicological studies

EGCG has potential neuroprotection against thrombin-associated toxicity in primary cortical neurons. It effectively eliminated thrombin-induced toxicity and stopped apoptosis by inhibiting JNK phosphorylation. It also may prevent thrombin-induced neuroapoptosis by preventing JNK activation [161]. A study showed the protective impact of EGCG against cadmium-induced cytotoxicity in rat brains. It inhibited mitochondrial dysfunction caused by Cd^2+^ and stopped lipid peroxidation. Its chelating and antioxidant properties likely contribute to its effectiveness in preventing lipid peroxidation and mitochondrial dysfunction caused by Cd^2+^ [162]. Another study showed a link between the neuroprotective effects of EGCG and the regulation of glutamate levels in a rat spinal cord organotypic culture. It effectively inhibited the glutamate transporter inhibitor, threohydroxyaspartate, thereby preventing glutamate excitotoxicity. It also may be a potential treatment option for ALS that includes glutamate excitotoxicity [163]. EGCG can reduce polyQ-mediated htt protein aggregation and cytotoxicity in vitro, potentially leading to the development of an innovative medication for HD and polyQ diseases [159]. Antioxidants and free radical scavengers can reduce the neurotoxicity of the Aβ protein, which is mediated by oxygen-free radicals. Antioxidants may reduce the risk of AD, making them a potential treatment option for the disease [164]. Moreover, a study investigated the neuroprotective effect of EGCG in the Aβ-induced neurotoxicity model. It increased the expression of γ-glutamylcysteine ligase, thereby strengthening the glutathione pool within cells [105]. A study found EGCG’s potential to modify insoluble Aβ and α-syn aggregates in a cell model system after discovering it suppresses fibrillogenesis in cell-free experiments. Altering their shape reduced the cytotoxic effects of mature Aβ and α-syn fibrils [165]. In addition, a study showed the potential of EGCG to mitigate the neurotoxic effects of formaldehyde by activating the Nrf2 signaling pathway [166]. EGCG decreased Aβ-induced neurotoxicity in cultured hippocampus neurons, reducing the harmful effects of oxygen free radicals [164]. Furthermore, EGCG effectively reduced inflammation and neurotoxicity in microglia and the hippocampus of rats and mice [167]. Another study investigated the efficacy of EGCG in mitigating BPA-induced neurotoxicity in the CA3 area of the rat hippocampus [168]. The neuroprotective effect of EGCG on PC12 cells against CORT-induced neurotoxicity is mediated through the stimulation of the Shh signaling system. EGCG has protective properties and activates the Shh signaling pathway in PC12 cells damaged by CORT. The Shh signaling pathway may play a role in the neuroprotective properties of EGCG against neurotoxicity caused by CORT [169]. Moreover, EGCG prevents AD by reducing the neurotoxicity associated with AD. It decreased neurotoxicity caused by Aβ by inhibiting endoplasmic reticulum stress-mediated apoptosis [170].

## Preclinical and clinical studies

In vivo tests on various animal models found the neuroprotective benefits of EGCG [48]. A study showed that intraperitoneal injection of 20 mg/kg of EGCG reduced Aβ levels and plaque formation in a transgenic mice model with “Swedish” mutant APP. The same group of researchers found that giving EGCG orally in drinking water at a dose of 50 mg/kg decreased the amount of Aβ deposited in the mutant mice [46]. Another study [171] showed transgenic mouse models of AD and the potential of EGCG (taken orally at a dose of 20 mg/kg/day for three months) to disrupt Aβ accumulation in several brain regions. Immunohistochemistry data demonstrated a 60% reduction in Aβ deposits in the frontal cortex and a 52% reduction in the hippocampus. In both areas, there was a decrease in the number of compact plaques labeled with Thioflavine-S histochemistry. Furthermore, compared to the control group, there was a decrease in the percentage of CD45, a marker of microglial activation, in the cortex (above 18%) and the hippocampus (28%). EGCG is used in the treatment of AD [95]. Moreover, a study found that fish oil and EGCG, when co-treated with N2a cells, enhance sAPP-alpha generation in a mouse model of AD. It can suppress cerebral Aβ deposition [172]. EGCG prevented the loss of neurons due to apoptosis and lipopolysaccharide (LPS) from impairing memory. It enhanced cytokine expression and inhibited LPS-induced astrocyte activation [49].

A study systematically reviewed GTC and its pure form, EGCG, in pre-clinical animal trials. Results showed that GTC extracts or pure EGCG had preventative effects on AD, improving learning and memory, potentially by reducing OS, Aβ plaque accumulation, and Tau protein phosphorylation [173]. Additionally, EGCG has been shown in numerous in vitro and pre-clinical investigations to have anti-inflammatory properties by modulating various molecular pathways. Research on AD syndrome showed that EGCG primarily reduces Aβ buildup by altering various biological processes. More preclinical research and well-planned clinical studies are needed to improve EGCG dose levels [93]. Preclinical research found that EGCG may have anti-amyloid properties and potentially prevent AD [96]. In 2019, EGCG was approved for a clinical trial on patients with multiple system atrophy, an uncommon NDs similar to PD, due to its proven effectiveness in preclinical models. EGCG supplementation did not significantly impact the progression of multiple system atrophy. It can cause hepatotoxic effects in some patients, advising against using doses exceeding 1200 mg [174]. Antioxidant therapy for non-alcoholic NDs has gained significant attention as a potential method to delay or mitigate OS-induced neurodegeneration. Clinical research found various antioxidants, such as EGCG. The integration of antioxidants from preclinical research into clinical settings faces several challenges. Clinical studies have shown limited positive results for any of the investigated antioxidants despite promising results in both in vitro and in vivo assays [175].

Furthermore, another study found the differences in EGCG levels are primarily due to its limited bioavailability. When EGCG is injected intravenously, it partially degrades before it reaches the intended tissues [176]. A study found that drinking more tea is linked to a lower risk of cognitive impairments. The study used data from 17 trials with 48,435 participants. Green tea consumption showed a significant inverse correlation, while black/oolong tea consumption did not. A dose–response meta-analysis showed a linear and adverse correlation between tea drinking and cognitive impairments [177]. Moreover, another study in Japan found that green tea may impact cognitive impairment. The study involved 33 residents in nursing homes with cognitive dysfunction. After a year of green tea consumption, there was no significant difference in MMSE-J score changes compared to the placebo group. However, the green tea group had significantly reduced levels of malondialdehyde-modified low-density lipoprotein, a measure of OS [14]. Furthermore, a study in Japan found that daily consumption of two or more cups of green tea (100 ml/cup) reduces the prevalence of cognitive impairment. Dementia, particularly AD, is less prevalent in Japan than in North America and Europe. Green tea consumption may impact cognitive performance, potentially impacting clinical and public health due to its minimal toxicity and lack of nutritional value [178].

## Conclusion and future perspectives

EGCG is a potential neuroprotective agent. It exhibits protective effects by reducing OS, inhibiting Aβ aggregation, modifying cell signaling pathways, and possessing anti-inflammatory properties. Furthermore, it also promotes autophagy and improves mitochondrial activity, supporting neuron survival and viability. Clinical research has shown that EGCG can effectively reduce neurodegenerative biomarkers and enhance cognitive function through supplementation. Despite positive results, EGCG’s therapeutic effectiveness necessitates determining bioavailability and appropriate dosage issues. The development of advanced delivery methods, such as nanoparticle-based formulations, aims to improve the stability and bioavailability of EGCG in the human body. Researchers are conducting extensive clinical trials to determine the optimal dosage schedules and long-term safety profiles of EGCG. Mechanistic studies aim to enhance our comprehension of the molecular basis of EGCG’s activity, specifically its impact on neuronal survival and function. Combination therapies aim to enhance EGCG’s neuroprotective efficacy by exploring its synergistic effects when combined with other treatments or lifestyle modifications. The study aims to conduct extensive clinical research to assess the long-term effects of EGCG administration on cognitive performance and the progression of NDs in patients. Future research in these areas could lead to the development of effective EGCG-based treatments, improved ND management, and extended life expectancy.

## Data Availability

No datasets were generated or analysed during the current study.
